# Fungal brain abscesses caused by *Acremonium* species

**DOI:** 10.1186/s43162-022-00183-z

**Published:** 2023-01-30

**Authors:** Hamdy Ibrahim, Mostafa ALfishawy, Attaa Ali, Safwat Abdel Maksod, Magdy Khorshed, Hanan Rady, Ahmed Alsisi, Adel Mohamed, Omar Alkassas, Marwa Haron, Suzan Saied

**Affiliations:** Intensive Care Unit Team, Embaba Fever Hospital, Giza, Egypt

**Keywords:** Fungal abscesses, *Acremonium*, COVID-19

## Abstract

Unusual fungal agents that exist environmentally as saprophytes can often lead to opportunistic infections, hyalohyphomycosis is a group of fungal infections caused by fungi characterized by hyaline septate hyphae and can infect both immunocompetent as well as immunocompromised patients, and *Acremonium* has drawn the attention of clinicians and microbiologists, as a potential pathogen in patients with and without underlying risk factors. It has also been increasingly implicated in systemic fungal diseases. Herein, we describe a case presentation of an immunocompromised patient with fungal brain abscesses due to *Acremonium* species.

## Background

*Acremonium* species are rarely described as opportunistic pathogens in humans. This case report presents brain abscesses caused by *Acremonium* species in a 64 years old Egyptian male, 2 weeks after falling in a water drain, He is a known diabetic, post-COVID-19, and 1 month before admission. He finished the last cycle of chemotherapy for Hodgkin lymphoma 6 months back before admission.

Stereotactic biopsy from the lesion and fungal culture revealed *Acremonium* species. At that time, the patient was mechanically ventilated and ultimately died. The purpose of this report is to alert physicians to such rare fungal infections as they are commonly resistant to most antifungal medications and also to highlight the importance of investigations for unknown microorganisms in patients with risk factors and the necessity of diagnosis for early adoption of specific treatment against such agents in order to reduce morbidity and mortality.

## Case presentation

A 62-year-old male known diabetic fell into a water drain while he was driving his car on a dark unpaved road. His head and face had many abrasions, bruises, and lacerations as did his extremities. His body was soaked all through with dirt. He was transferred to the nearest hospital, he was not in shock, and his consciousness level and mental function were intact; after suturing the wounds, he was admitted 24 h for observation and discharged on antibiotics and daily dressing. After 1 week, he developed a fever and productive cough of a moderate amount of sputum, yellowish in color not related to posture, and with no hemoptysis. He is a known diabetic and had a history of Hodgkin’s lymphoma and finished his last cycle of chemotherapy 6 months before admission, and he suffered from COVID-19 infection 1 month before this accident, and fortunately, he recovered without complications.

On physical examination, he had a temperature of 38 °C with blood pressure of 130/70 mmHg, pulse of 85/min, and a respiratory rate of 25/min. Neck veins were not congested. No jaundice, pallor, cyanosis, or edema of lower limbs and there was no lymphadenopathy.

Again he was admitted and routine laboratory investigations were requested which had no significant abnormality.

Chest: scattered crackles bilaterally with normal heart and abdominal examination. He received antibiotics levofloxacin 500 mg once for 5 days, ceftazidime, and metronidazole. A few days later he suffered from mild dizziness, severe bursting headache, and generalized seizures followed by loss of consciousness.

**Fig. 1:**
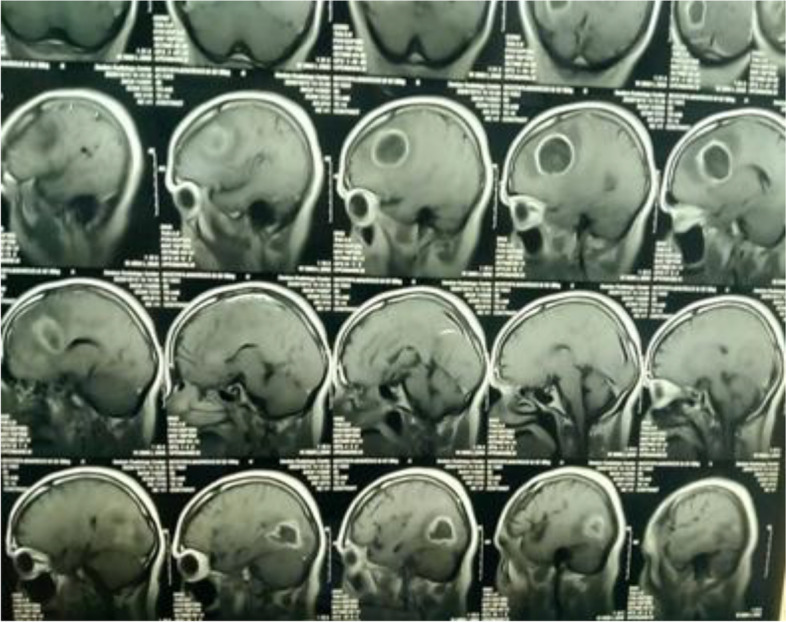
MRI with contrast, sagittal section showing ring-enhancing lesions in both frontal and parietal lobes

**Fig. 2:**
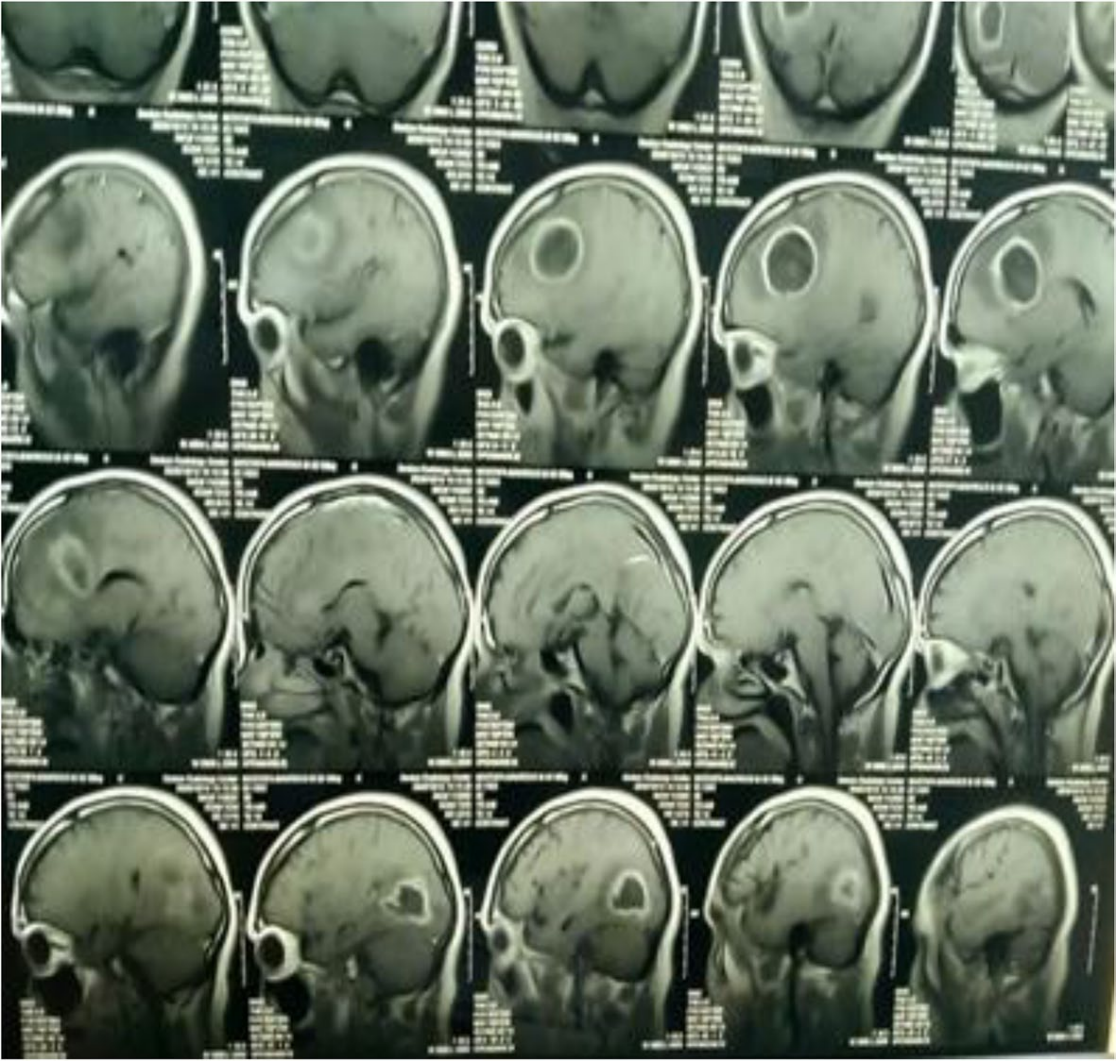
MRI with contrast sagittal and coronal sections show left frontal and right parietal—intra-axial hypodense brain lesions with a margin of enhancement

**Fig. 3:**
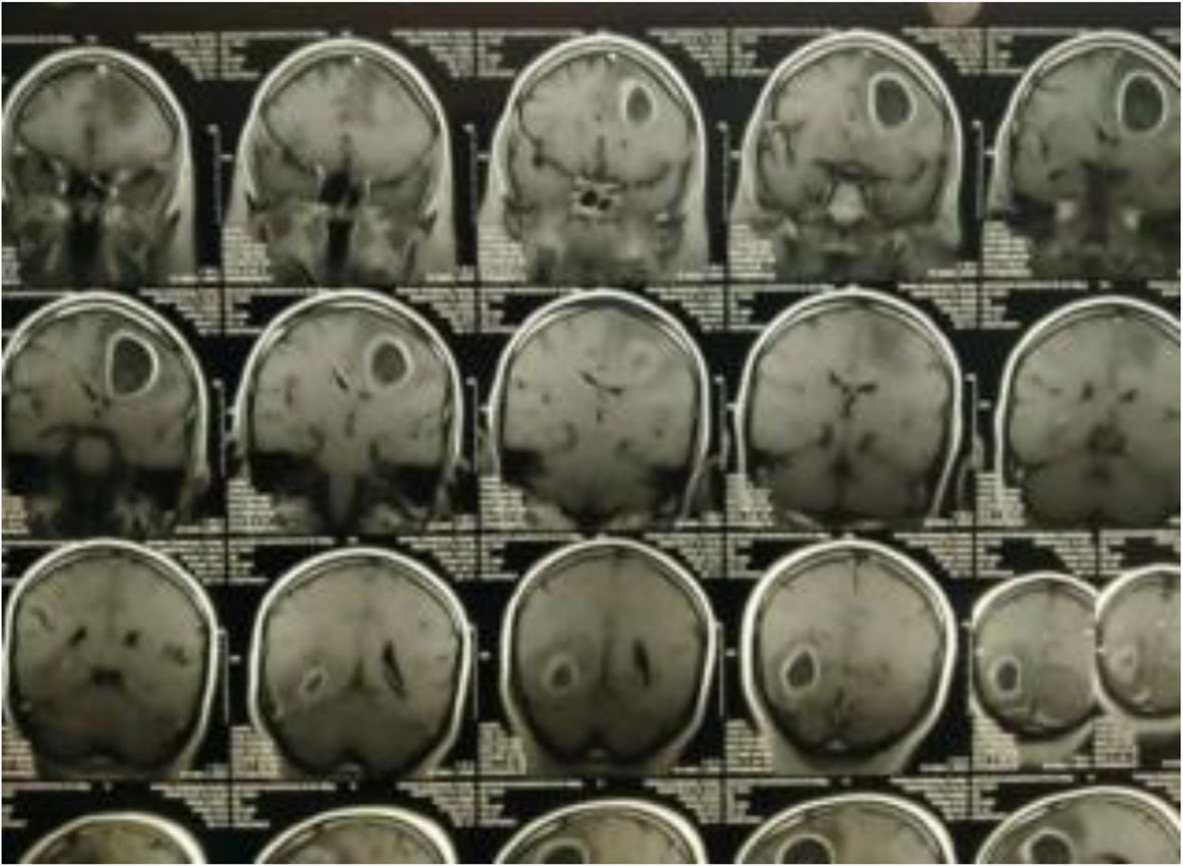
MRI with contrast sagittal and coronal sections show left frontal and right parietal—intra-axial hypodense brain lesions with a margin of enhancement

**Fig. 4:**
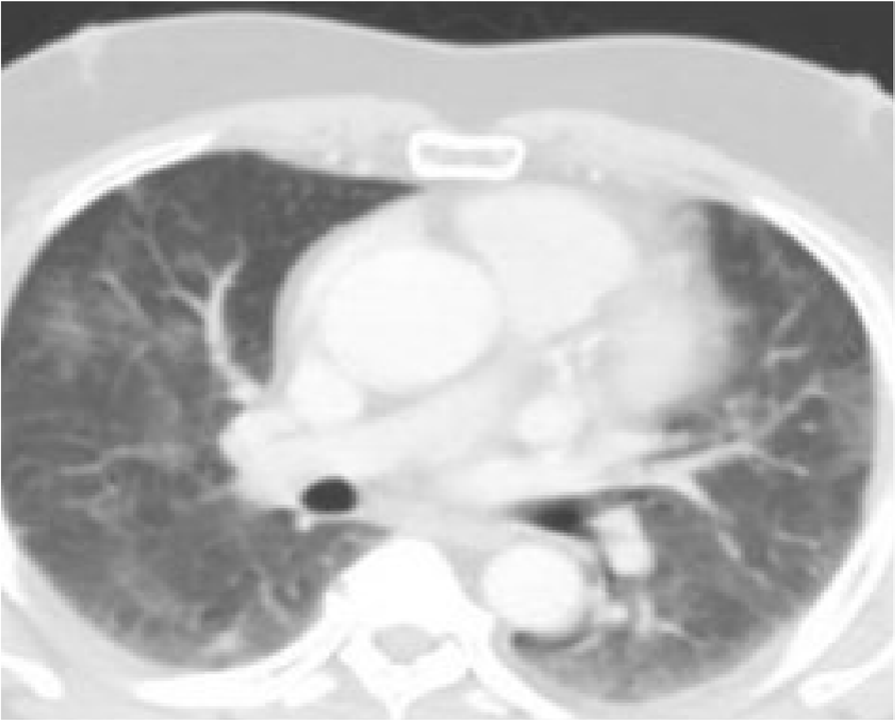
CT chest: bilateral multifocal patchy areas of ground glassing associated with reticulations and fibroelastic bands, probably post-COVID-19 pulmonary fibrosis (sequelae of post-COVID-19 viral infection)

MRI revealed multiple brain abscesses (Fig. 1), he was referred for PET/CT evaluation, and PET images from the skull vault to the mid-thigh were obtained and revealed the following: left frontal and right parietal—intra-axial hypodense brain lesions with a margin of enhancement and increased metabolic activity (Figs. 2 and 3), they are mostly brain abscesses, bilateral multifocal patchy areas of ground glassing associated with reticulations and fibroelastic bands, probably post COVID-19 pulmonary fibrosis (sequelae of COVID-19 viral infection) (Fig. 4).

A stereotactic biopsy brain revealed no result of culture and pathology.

Gram stain shows many pus cells, culture: no growth detected after aerobic and anaerobic incubation. The sample was sent for fungal culture and sensitivity. Considering the history of being immunocompromised and recent falling in a dirty water drainer, a fungal brain abscess was the suspected diagnosis. The report for fungal culture and sensitivity revealed *Acremonium.* species, which is sensitive to amphotericin B, caspofungin, and fluconazole but resistant to flucytosine. The patient was managed with combined amphotericin B and fluconazole, but unfortunately, he did not survive and was declared dead after 1 week of his ICU admission on the mechanical ventilator.

### Diagnosis

**Fig. 5:**
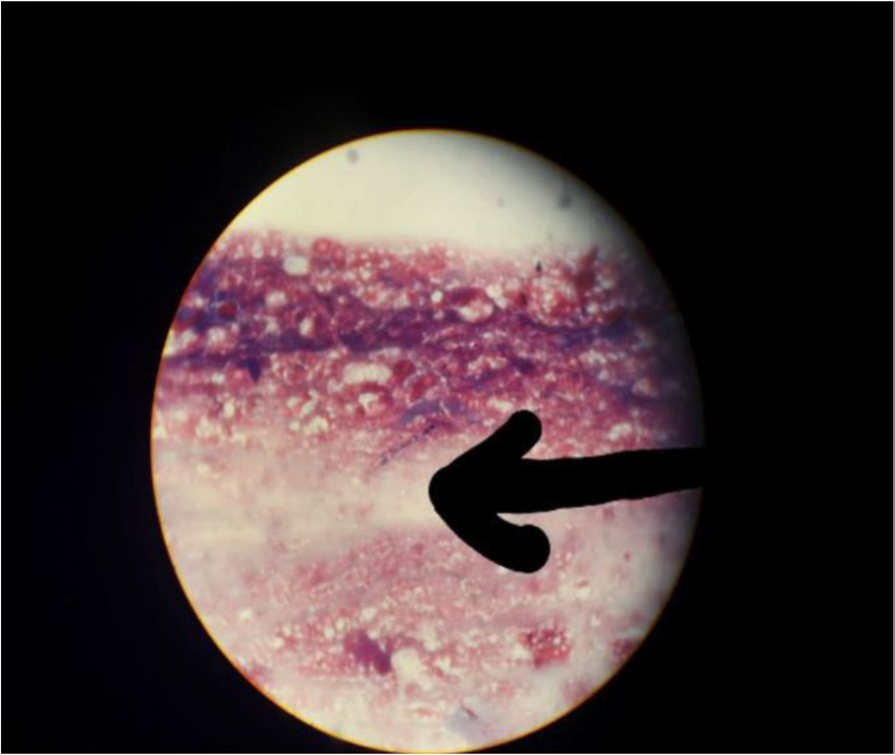
Mold fungi *Acremonium* by tissue culture (stereotactic brain biopsy for fungal culture)

Fungal brain abscesses due to *Acremonium* species (Fig. 5).

## Discussion

Brain abscesses are serious and potentially life-threatening emergency that requires prompt diagnosis and treatment. Any delay in the management of brain abscesses can lead to poor health outcomes. Both pharmacological and surgical treatment may prove to be very significant in managing fungal brain abscesses [1, 2]. However, given the rarity of *Acremonium* infection and the limited published literature available, there are no established guidelines regarding its treatment. Also, the rarity of cases involving this microorganism justifies the presentation of this case.

A hyalohyphomycosis is a group of fungal infections caused by fungi characterized by hyaline septate hyphae and can infect both immunocompetent as well as immunocompromised patients. Species of *Acremonium* are common in the environment, and several have been described as causing invasive diseases in humans [3]. Infections in humans typically develop following traumatic inoculation of the fungus with keratitis and mycetoma being the most common. Locally invasive infections such as osteomyelitis, sinusitis, and peritonitis and less frequently central nervous system infections have been reported. Neurofungal infections, meningitis, or abscesses are associated with extremely high mortality caused by delayed onset of therapy, severe underlying disease, and multi-resistant fungal organisms [4]. *Acremonium* has drawn the attention of clinicians and microbiologists, as a potential pathogen in patients with and without underlying risk factors [5]. There is little information regarding the susceptibility of *Acremonium* species to antifungals, but they are characteristically resistant to anti-*Candida* agents, fluconazole, and flucytosine [6]. Minimum inhibitory concentrations (MICs) against amphotericin B are commonly elevated which suggests the poor activity of this drug. *Acremonium sclerotigenum* and *Acremonium egypticum* group seem to be less susceptible to amphotericine B [7]. Newer azoles such as voriconazole and posaconazole may be effectively used against this fungus [8]. However flucytosine, echinocandins, and fluconazole are not active against it [9]. Posaconazole and voriconazole may be the best therapeutic alternative [10].

Risk factors in this immunocompromised patient include his age, previous COVID-19 infection, diabetes mellitus, malignancy, and prolonged use of antibiotics. To the best of our knowledge, there is only one case report of brain abscess caused by *Acremonium* species (recorded in an 18-year-old male in Pakistan (Anis et al.) [11] and our case is the second case.

## Conclusion

Fungal infection should be considered in the differential diagnosis of brain abscesses, especially in immune-suppressed patients. Although the outcome is frequently fatal in patients with fungal brain abscess, delay in the diagnosis also contributes to the high mortality. Early diagnosis, aggressive surgical procedures, and antibiotic therapy for fungal brain abscesses may reduce morbidity and mortality. Unusual environmental contaminant fungi like *Acremonium* species can cause severe and fatal fungal infections in the immune suppressed. It is essential for infectious disease specialists, neurosurgeons, and microbiologists to follow a multidisciplinary approach to the effective delivery of care in cases of rare life-threatening fungal infections involving the CNS *Acremonium*. The management of different antifungals in various clinical situations has been very conflicting and hence needs to be carefully evaluated. Clinicians must be aware of the list of fungal pathogens resistant to these drugs among which *Trichosporan*, *Aspergillus ustus*, and *Acremonium* species are predominant.

## Abbreviations

COVID: Coronavirus disease
MRI: Magnetic resonance imaging
PET/CT: Positron emission tomography/computerized tomography
MIC: Minimum inhibitory concentration
